# Organosilane-Based Coating of Quartz Species from the Traditional Ceramics Industry: Evidence of Hazard Reduction Using *In Vitro* and *In Vivo* Tests

**DOI:** 10.1093/annweh/wxx014

**Published:** 2017-02-28

**Authors:** Christina Ziemann, Alberto Escrig, Giuliana Bonvicini, Maria Jesús Ibáñez, Eliseo Monfort, Arturo Salomoni, Otto Creutzenberg

**Affiliations:** 1 Fraunhofer Institute for Toxicology and Experimental Medicine ITEM, Nikolai-Fuchs-Str. 1, 30625 Hannover, Germany;; 2 Instituto de Tecnología Cerámica–AICE, Universitat Jaume I, Campus Universitario Riu Sec, Avenida Vicent Sos Baynat, 12006 Castellón, Spain;; 3 Centro Ceramico di Bologna (CCB), Via Martelli, 26, 40138 Bologna, Italy

**Keywords:** coating of quartz, organosilane, quartz toxicity, rat alveolar macrophages, respirable crystalline silica, silanol groups, silicosis, traditional ceramics industry

## Abstract

The exposure to respirable crystalline silica (RCS), e.g. quartz, in industrial settings can induce silicosis and may cause tumours in chronic periods. Consequently, RCS in the form of quartz and cristobalite has been classified as human lung carcinogen category 1 by the International Agency for Research on Cancer in 1997, acknowledging differences in hazardous potential depending on source as well as chemical, thermal, and mechanical history. The physico-chemical determinants of quartz toxicity are well understood and are linked to density and abundance of surface silanol groups/radicals. Hence, poly-2-vinylpyridine-*N*-oxide and aluminium lactate, which effectively block highly reactive silanol groups at the quartz surface, have formerly been introduced as therapeutic approaches in the occupational field. In the traditional ceramics industry, quartz-containing raw materials are indispensable for the manufacturing process, and workers are potentially at risk of developing quartz-related lung diseases. Therefore, in the present study, two organosilanes, i.e. Dynasylan® PTMO and Dynasylan® SIVO 160, were tested as preventive, covalent quartz-coating agents to render ceramics production safer without loss in product quality. Coating effectiveness and coating stability (up to 1 week) in artificial alveolar and lysosomal fluids were first analysed *in vitro*, using the industrially relevant quartz Q1 as RCS model, quartz DQ12 as a positive control, primary rat alveolar macrophages as cellular model system (75 µg cm^−2^; 4 h of incubation ± aluminium lactate to verify quartz-related effects), and lactate dehydrogenase release and DNA strand break induction (alkaline comet assay) as biological endpoints. *In vitro* results with coated quartz were confirmed in a 90-day intratracheal instillation study in rats with inflammatory parameters as most relevant readouts. The results of the present study indicate that in particular Dynasylan® SIVO 160 (0.2% w/w of quartz) was able to effectively and stably block toxicity of biologically active quartz species without interfering with technical process quality of certain ceramic products. In conclusion, covalent organosilane coatings of quartz might represent a promising strategy to increase workers’ safety in the traditional ceramics industry.

## Introduction

Prolonged exposure to respirable crystalline silica (RCS) can induce silicosis and may cause lung tumours in chronic periods. In 1997, the International Agency for Research on Cancer (IARC) therefore classified RCS, in the form of quartz or cristobalite, as category 1 human carcinogen. But, differences in the hazardous potential of RCS were acknowledged, depending on its source (inherent characteristics), chemical, thermal, and mechanical history and the specific industrial scenario. Namely, certain external factors/events can lead to activation or passivation of biologically active RCS surfaces, resulting in ‘chemical agents’ with the same nominal composition (SiO_2_), but with radically opposed toxicities (Fubini, 1998a, 1998b).

Schlipköter and Brockhaus (1961) postulated that silanol groups (Si-OH), abundant on the quartz surface and also generated mechanically and by hydrolysis of siloxane bridges (Si-O-Si) (Nolan *et al.*, 1981), represent, besides surface radical formation (Fubini, 1998a, 1998b), the main determinants of RCS-mediated pathological effects. By using as-grown quartz crystals with intact surfaces, Turci *et al.* (2016) recently demonstrated that the biological activity of quartz thereby seems not be determined by silanol groups *per se* but that it needs conchoidal surface fractures and thus a disordered panel of silanols, siloxanes, and rings for quartz–cell membrane interactions. But, it should in any case be possible to neutralize these active centres and to reduce silanol heterogeneity by strong adsorption of certain molecules such as aluminium salts, metal iron, proteins, or phospholipids to prevent deleterious quartz–cell membrane interactions, which can result in formation of reactive oxygen species, membrane damage/haemolysis, cytotoxicity, DNA damage, cytokine release, and subsequently lung toxicity, inflammation, silicosis, and even tumour formation (Fubini, 1998a, 1998b; Schins *et al.*, 2002; Albrecht *et al.*, 2004). Interestingly, clear correlation between quartz-mediated haemolytic activity and inflammasome activation could be demonstrated by Pavan *et al.* (2014), pointing to the same physico-chemical structures as determinants.

Polyvinylpyridine-*N*-oxide (PVPNO) and aluminium salts have already been tested as non-covalent surface modifications to treat or prevent silicosis, however, with limited success (Weller, 1975; Zhao *et al.*, 1983; Prügger *et al.*, 1984; Bégin *et al.*, 1986; Goldstein and Rendall, 1987; Dubois *et al.*, 1988; Dufresne *et al.*, 1994; Bégin *et al.*, 1995). PVPNO forms strong hydrogen bonds via NO^−^ groups, thus masking negatively charged SiO^−^ groups (reviewed by Castranova, 1995), whereas aluminium lactate (AL) can block surface charges and radical formation via the aluminium cation and related ionic forces (Bégin *et al.*, 1986; Brown *et al.*, 1989; Duffin *et al.*, 2001; Albrecht *et al.*, 2004, 2007). AL seems to be less effective *in vivo* than PVPNO, perhaps because of different modes of action and differential modification of cell type-specific uptake and elimination of treated quartz by alveolar macrophages (AM) (Höhr *et al.*, 2002; Albrecht *et al.*, 2007, 2009). Irrespective of non-covalent binding, however, PVPNO was able to clearly reduce NLRP3 inflammasome activation by quartz DQ12 in the rat lung, using IL-1β and caspase-1 immunostaining as readouts (Peeters *et al.*, 2014). Unlike PVPNO and AL, organosilane compounds, which were already shown to attenuate quartz toxicity *in vitro* and *in vivo* (Wiessner *et al.*, 1990; Vallyathan *et al.*, 1992; Castranova *et al.*, 1995), react chemically with Si-OH groups, leading to potentially more durable, covalent, thin quartz surface coatings.

Though the research line on passivation of reactive quartz surfaces was already started in 1960, mainly for therapeutic purposes (Weller, 1975; Zhao *et al.*, 1983; Prügger *et al.*, 1984; Dubois *et al.*, 1988), none of the mentioned coating agents appear to have been used so far preventively on an industrial scale to increase workers’ safety. Only Vallyathan *et al.* (1991) proposed to incorporate organsilanes into water sprays of drill bits. To close this gap, the EU-funded project SILICOAT was set up, which aimed at increasing workers’ safety in the traditional ceramics’ industry (tiles, tableware, sanitary ware) by developing and implementing cost-effective, covalent RCS-coating technologies into ceramic processes. RCS-containing materials are indispensable for ceramics’ production, and many workers are thus potentially at risk. The coatings should stably saturate reactive surface Si-OH groups and inhibit quartz-specific toxicity, while maintaining product quality.

As an integral part of the SILICOAT project, the present study primarily aimed at biologically analysing and proofing *in vitro* and *in vivo* quartz-coating effectiveness and stability of two different organosilane-based quartz coatings. Organosilanes are widely used industrially in treating/functionalizing siliceous (Plueddemann, 1991) and metal surfaces and are available at affordable prices.

## Methods

### Model quartz

The industrial quartz Q1, used in a sanitary ware composition, was selected as model quartz for the coating trials. Crystalline silica content (100% w/w) was determined by X-ray diffraction (Philips 1820/00 diffractometer, The Netherlands), particle size distribution (*d*_10_ = 1.9, *d*_50_ = 12.1, and *d*_90_ = 37.2 µm) by laser diffraction (Malvern Mastersizer 2000, UK), and surface area (0.87 m^2^ g^−1^) by the Brunauer–Emmett–Teller method (TriStar 3000, Micromeritics, USA).

### Negative and positive controls

For both *in vitro* and *in vivo* experiments, highly active quartz, DQ12 midsize Dörentrup (87% α-quartz, 13% amorphous silica; geometric mean diameter, weighted by mass: 3.0 µm; Bergbauforschung, Essen, Germany), served as technical/methodological positive control for quartz-dependent biological effects. DQ12 is a very active and stable quartz dust, showing no aging effect (i.e. loss of toxic potency with time). It is the number 1 quartz in a ranking list of the most active quartzes. Therefore, DQ12 has become the standard reference positive control quartz for experimental biology in Europe (Robock, 1973; Clouter *et al*., 2001; Creutzenberg *et al.*, 2008). Water-sized aluminium oxide (Al_2_O_3_; geometric mean diameter, weighted by mass: 4.1 µm), derived from fused, active, neutral Al_2_O_3_, type 507 C (Sigma–Aldrich, Taufkirchen, Germany) served as *in vitro* particulate negative control. Al_2_O_3_ was relatively inert in previous short-term assays with cultured rat AM (Ziemann *et al.*, 2009, 2014a, b). As non-particulate *in vitro* and *in vivo* negative controls, cell culture medium and physiological saline (0.9%) were used, respectively.

### Coating agents and quartz-coating procedures

**Figure 1.**
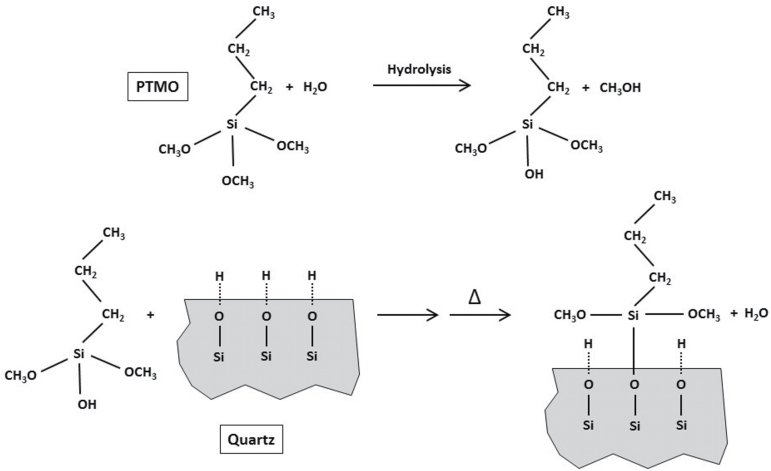
Hydrolysis of PTMO and coating reaction with reactive silanol groups on the quartz surface.

As coating agents, two commercially available organosilanes were selected with help of Evonik Degussa International AG (Essen, Germany), i.e. hydrophobic Dynasylan® PTMO [propyltrimethoxysilane (PTMO); Fig. 1] and hydrophilic Dynasylan® SIVO 160 (SIVO160; oligomeric, amino-modified siloxane), to have clearly varying technical options in the industrial implementation trials. For quartz coating, PTMO was first suspended in water. To accelerate initial hydrolysis 0.01 M hydrochloric acid was added (HCl:PTMO ratio by mass of 1:2.5). Liquid was stirred until homogeneity, before Q1 was added and allowed to react with hydrolysed PTMO (0.1, 0.5, or 1.4% w/w of quartz) under stirring for 3 h at room temperature. SIVO160-coated Q1 was prepared similarly, but without the hydrolysis step, as SIVO160 represents a stabilized, pre-hydrolysed suspension. SIVO160 (0.2, 0.4, or 1.0% w/w of quartz) was incubated with Q1 for 2 h. All quartz suspensions were finally filtered using Büchner flasks and then repeatedly washed with ethanol to remove any surplus of additive. Uncoated reference Q1 particles were treated equally, but without organosilane addition. The coating procedures were initially developed under laboratory-scale conditions, before being adapted to production plant conditions.

### Endotoxin content and sizing of particle fractions

To avoid biological test artefacts, endotoxin content of all investigated particles was initially determined, using the ‘limulus amebocyte lysate (LAL) assay’ (Lonza, Cologne, Germany), and was shown for all samples to be clearly below the detection limit or below 8 ng rat^−1^ lung, as threshold for *in vivo* relevance (Elder *et al.*, 2000). Whereas *in vitro* experiments were performed with the bulk materials, water-sizing was done for *in vivo* use. For this, 2.5 or 2 × 2.5 g of bulk material were added to a ~10 cm water column (volume ~110 ml) in a glass container. After sedimentation for 15–35 min, 90–95 ml of supernatant were freeze-dried using stainless steel dishes. Particle size distribution was checked by scanning electron microscopy. Geometric mean diameters, weighted by mass, were 4.17 (Q1), 4.49 (0.5% PTMO), and 4.85 µm (0.2% SIVO160).

### Cell isolation, cell culture, and particle treatment *in vitro*

Primary rat AM, as main regulators of inflammation and clearance in the lung, were used as *in vitro* model. AM were previously shown to be highly quartz-responsive after *in vitro* exposure (Ziemann *et al.*, 2009). AM were isolated from pathogen-free female Wistar rats [strain: Crl:WI(Han); Charles River, Sulzfeld, Germany] by bronchoalveolar lavage (BAL) with physiological saline. About 1.5–2 × 10^5^ cells per well were plated in hydrophobic 24-well plates (NUNC, Wiesbaden, Germany) and pre-cultured for 24 h, as described previously (Ziemann *et al.*, 2014b). Twofold concentrated particle stock suspensions were prepared in cell culture medium without FCS and antibiotics by ultrasonication for 5 min in a water bath. To avoid cell activation, particles were added to the cultures (final concentration: 75 µg cm^−2^) without medium exchange. AM were incubated for 4 h in the dark at 37°C and 5% CO_2_ in a humidified atmosphere. One of two parallel cultures additionally received 100 µM AL (Aldrich, Taufkirchen, Germany) to verify quartz-specific effects. The quartz-quenching effect of AL had previously been confirmed in AM (Monfort *et al.*, 2008; Ziemann *et al.*, 2009, 2014a). After incubation, supernatants were taken for determination of lactate dehydrogenase (LDH) activity. For gentle cell detachment, needed for the alkaline comet assay, AM were then placed on ice for 10 min. For the LDH assay, a ‘background control’ (wells without cells, but with culture medium) and a ‘maximum control’ (cells treated for 10 min with 1% Triton X-100 = maximum amount of releasable enzyme activity) were included.

### LDH assay *in vitro*

LDH release/activity, indicating disturbance of cell membrane integrity, was chosen as *in vitro* cytotoxicity endpoint. This endpoint had previously been used to compare different quartz-containing ceramic dusts in AM (Ziemann *et al.*, 2009), and was additionally used in the present study as early *in vivo* biomarker in BAL fluid. LDH activity was determined in culture supernatants using the ‘Cytotoxicity Detection Kit’ (Roche, Mannheim, Germany). Absorbance was measured in triplicate at 490 and 630 nm with an ELISA reader. Percent LDH activity was finally calculated by subtracting the ‘background control’ absorbance value and setting the ‘maximum control’ value to 100%.

### Alkaline comet assay *in vitro*

To detect direct DNA damage, the alkaline comet assay was used (Singh *et al.*, 1988; Tice *et al.*, 2000). This test had previously demonstrated good quartz DQ12 responsiveness, with complete inhibition by parallel AL treatment (Ziemann *et al.*, 2009, 2014a). Detailed methodology had been described previously (Ziemann *et al.*, 2014b). All methodological steps following cell detachment were done under red light to avoid unspecific DNA damage. Coded slides were semi-automatically analysed using an Axioskop fluorescence microscope (Zeiss, Göttingen, Germany) and the Comet Assay III software (Perceptive Instruments, Bury St. Edmunds, UK). Tail intensities (TI) of 100 nuclei were determined per slide/treatment and experiment, precluding so-called ‘hedgehogs’ or overlapping nuclei/comets from analysis. For every question, three independent experiments were performed. Means ± standard deviation (SD) were finally calculated from the mean TIs of the independent experiments.

### Artificial physiological fluids and *in vitro* testing of coating stability

Coating stability of 0.5% PTMO-coated Q1, showing the best *in vitro* results, was tested for up to 1 week in both artificial alveolar fluid (AAF; pH 7.4) and lysosomal fluid (ALF; pH 4.5; representing macrophages’ lysosomal ambience). AAF was prepared as described in detail by Kirkland *et al*. (2015). ALF preparation resembled that described by Hedberg *et al*. (2010), with slight modifications. Na_2_HPO_4_ content was 0.095 g l^−1^, and 2.3 ml of a 16% (w/w) formaldehyde stock solution were added. For both AAF and ALF preparation HPLC grade, ultrapure water and salts of highest purity were used. For testing of coating stability 250 mg of Q1 or 0.5% PTMO-coated Q1 were incubated in 40 ml of AAF or ALF in a drying chamber at 37°C for 24, 48, 72, or 168 h. Tubes were rotated at low frequency to avoid sedimentation and to enable good particle wetting. After incubation, particles were washed 3× to remove free coating material. To this, samples were centrifuged (10 min, 1932 g), supernatant was discarded, and particles were resuspended in ultrapure water. After washing, samples were resuspended in 25 ml of water, transferred to glass staining tanks, and freeze-dried. As references (0 h), coated and uncoated Q1 samples, not incubated with AAF or ALF, but subjected to the whole washing and freeze-drying procedure were used to minimize differences in sample activity from dissimilar handling. Finally, cytotoxicity and genotoxicity of the AAF- and ALF-treated samples were determined *in vitro*.

### *In vivo* validation study

To finally investigate coating stability *in vivo*, a 90-day study with five treatment groups was set up: (i) physiological saline, (ii) Q1, (iii) Q1 + 0.5% hydrolysed PTMO w/w of quartz, (iv) Q1 + 0.2% SIVO160 w/w of quartz, and (v) quartz DQ12. This study was performed in compliance with the German Federal Act on the Protection of Animals (2013). Male Wistar rats [strain: Crl:WI(Han); Charles River] were randomized into groups of five animals. Animal age was ~7 weeks at delivery and ~9 weeks at treatment start. Particles (total dose 1 mg animal^−1^) were administered to the lungs by intratracheal instillation of two 0.5 mg aliquots in 0.3 ml of physiological saline on consecutive days. To minimize particle agglomeration and assure homogenous distribution in the alveolar region, application suspensions were ultrasonicated and shaken moderately prior to instillation. On day 28 or 90 post-instillation, rats were sacrificed after anaesthesia with an overdose of pentobarbital sodium (Narcoren™) by cutting the *vena cava caudalis*. BAL was subsequently performed using the method of Henderson *et al.* (1987) with minor modifications. Lungs were lavaged twice with 5 ml of 0.9% NaCl each. BAL fluid was collected in calibrated tubes on ice and harvested volumes were recorded. Study endpoints comprised body weights (before treatment, at weekly intervals in the post-treatment observation period, terminally), terminal lung wet weights, and total leukocyte counts in the BAL fluid. Total leukocytes were counted light microscopically using a Fuchs-Rosenthal counting chamber (unit: cells per millilitre of lavagate). Differential cell counts were assessed by preparing two cytoslides (cytocentrifuge, Shandon Co., Frankfurt, Germany) and microscopically analysing 400 leukocytes per rat regarding macrophage, polymorphonuclear neutrophil (PMN; unspecific inflammation) and lymphocyte (specific inflammation) numbers. Results were given in absolute numbers per BAL fluid volume. After centrifugation of the BAL fluid, biochemical indicators, relevant for diagnosis of lung damage, i.e. LDH release (membrane damage), β-glucuronidase activity (phagocytic activity and/or lysis of macrophages), and total protein levels (transudation and epithelial cell damage) were determined in supernatants. All biochemical parameters were analysed according to routine clinical chemistry protocols using a Cobas Fara device (Roche Co., Grenzach, Germany).

### Statistics

In the *in vitro* experiments, statistically significant differences from the particulate negative control were calculated by Student’s *t*-test for unpaired values (two tailed), whereas Student’s *t*-test for paired values (two tailed) was used to evaluate differences between uncoated and coated and between AL-untreated and AL-treated samples. The *in vivo* tests were statistically analysed using Dunnett’s test (treated groups versus negative control) and *t*-test for unpaired values (two-tailed, single treatment groups versus control and uncoated versus coated quartz). Both *in vitro* and *in vivo* data were considered significant, if *P* ≤ 0.05. For statistical analyses, the Sigma Stat 3.1® software package (Systat Software, Inc., Point Richmond, CA, USA) was used.

## Results

### *In vitro* determination of coating efficiency— laboratory scale

**Figure 2.**
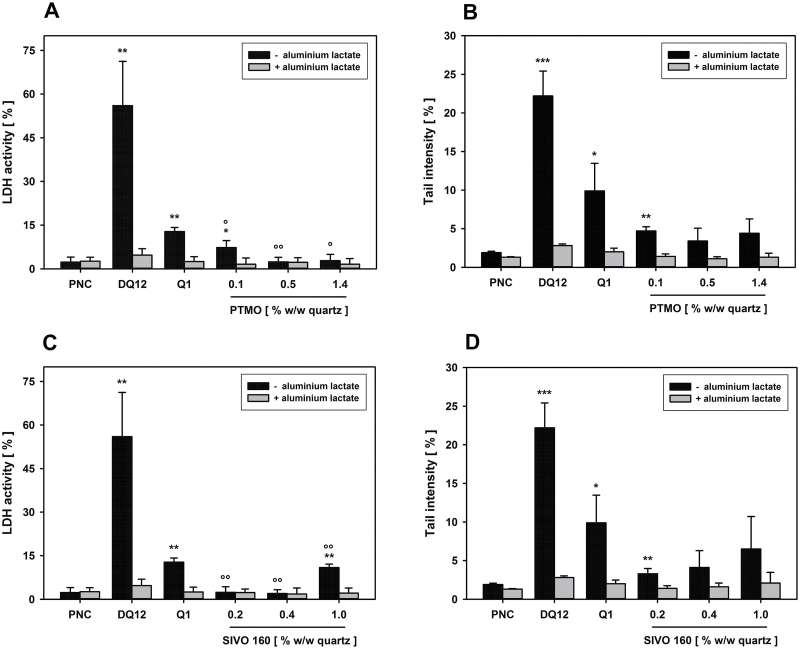
*In vitro* determination of coating effectiveness. AM were incubated ± 100 μM AL for 4 h with 75 μg cm^−2^ of uncoated Q1 or Q1, coated with different amounts of hydrolysed PTMO (A and B) or SIVO160 (C and D), Al_2_O_3_ as particulate negative (PNC) or DQ12 as quartz positive control. LDH release (A and C) and the alkaline comet assay (B and D) were used to detect membrane and direct DNA damage, respectively. Data represent means ± SD of three independent experiments. */**/***Significantly different from PNC: *P* ≤ 0.05/0.01/0.001, respectively, Student’s *t*-test for unpaired values, two tailed. °/°°/°°°Significantly different from uncoated Q1: *P* ≤ 0.05/0.01/0.001, respectively, Student’s *t*-test for paired values, two tailed.

**Figure 3.**
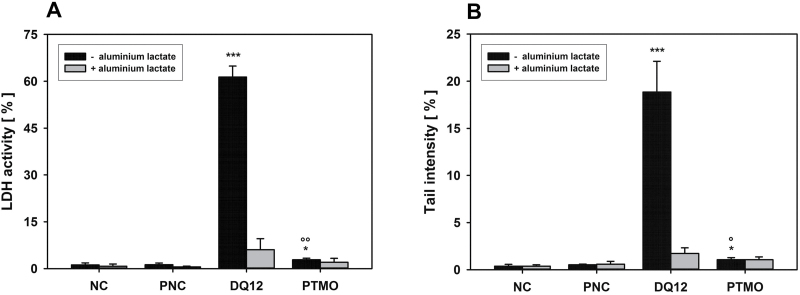
*In vitro* determination of coating effectiveness using the quartz positive control DQ12. AM were incubated ± 100 μM aluminium lactate for 4 h with 75 μg cm^−2^ of uncoated quartz DQ12 or DQ12, coated with 0.5% hydrolysed PTMO (w/w quartz), culture medium as negative (NC), or Al_2_O_3_ as particulate negative control (PNC). LDH release (A) and the alkaline comet assay (B) were used to detect membrane and direct DNA damage, respectively. Data represent means ± SD of three independent experiments. */***Significantly different from PNC: *P* ≤ 0.05/0.001, respectively, Student’s *t*-test for unpaired values, two tailed. °/°°Significantly different from the uncoated DQ12 sample: *P* ≤ 0.05/0.01, Student’s *t*-test for paired values, two tailed.

Q1 was coated with hydrolysed PTMO or SIVO160 under laboratory-scale conditions, using different amounts of the coating agents. Both PTMO and SIVO160 concentration dependently quenched Q1-induced LDH release. Hydrolysed PTMO was most effective at 0.5 and 1.4% w/w of quartz. Complete inhibition of LDH release by SIVO160 was noted at 0.2 and 0.4% w/w (Figs 2A and C), whereas 1.0% SIVO160 was substantially less potent. Both PTMO and SIVO160 clearly reduced Q1-enhanced mean TI and thus direct DNA damage. Nevertheless, slight quartz-dependent effect persisted, as determined by parallel AL incubation. DNA damage was maximally inhibited with the 0.5% PTMO (81% inhibition) and 0.2% SIVO160 (83% inhibition) coatings, but did not reach statistical significance (high variability in mean TI of pristine Q1). Based on highest quenching efficiencies, subsequent experiments were performed with 0.5% PTMO and 0.2% SIVO160. The toxicity-reducing potential of the organosilane coatings was proven with 0.5% PTMO-coated quartz DQ12. As observed for Q1, a 0.5% PTMO coating significantly inhibited (by 97%) both the strong DQ12-induced increase in LDH release and induction of direct DNA damage (Fig. 3).

### *In vitro* determination of coating stability

**Figure 4.**
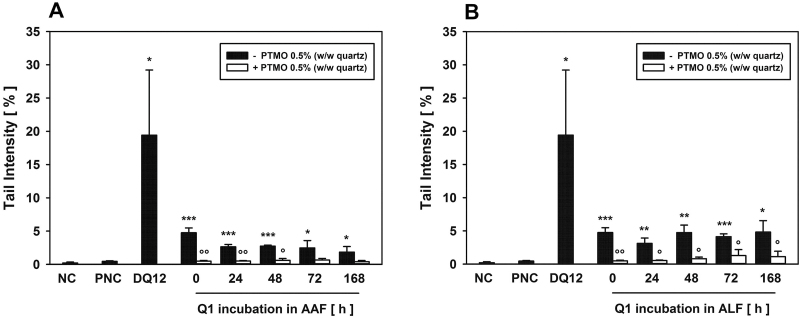
*In vitro* determination of coating stability using the alkaline comet assay. Pristine Q1 and Q1 coated with 0.5% hydrolysed PTMO (w/w quartz) were incubated for 0, 24, 48, 72, or 168 h in (A) AAF or (B) ALF and were subsequently washed three times and finally freeze-dried. Primary rat alveolar macrophages were then incubated for 4 h at 37°C with the different quartz samples or culture medium as negative (NC), Al_2_O_3_ as particulate negative (PNC), and DQ12 as quartz positive control, using a concentration of 75 μg cm^−2^ and subjected to alkaline comet assay analyses. Data represent means ± SD of three independent experiments. */**/*** Significantly different from Al_2_O_3_: *P* ≤ 0.05/0.01/0.001, respectively, Student’s *t*-test for unpaired values, two tailed. °/°°Significantly different from uncoated Q1: *P* ≤ 0.05/0.01, respectively, Student’s *t*-test for paired values, two tailed.

Coating stability was investigated *in vitro* by incubating pristine/uncoated and 0.5% PTMO-coated Q1 in AAF or ALF for up to 168 h at 37°C. DNA damage, as the most sensitive endpoint, was used as readout. Pristine Q1, incubated in AAF, significantly induced DNA damage, however, reduction in activity was noted with time (mean TI fresh Q1: 4.8 ± 0.7% and after 168 h: 1.8 ± 0.8%). After 0, 24, and 48 h of incubation in AAF, 0.5% PTMO-coated Q1 induced significantly less DNA damage than uncoated Q1. After 72 and 168 h quenching of genotoxicity was still obvious, but did not reach statistical significance. All mean TI values for coated Q1 were within or slightly above the range for the particulate negative control Al_2_O_3_, indicating good coating stability at pH 7.4 (Fig. 4A). After incubation in ALF (pH 4.5), pristine Q1 predominantly demonstrated mean TIs like fresh Q1 (Fig. 4B). Mean TI was maximal after 168 h of incubation (4.8 ± 1.7%), compared to 0.5 ± 0.1% for Al_2_O_3_. At all incubation times, the DNA-damaging potential of 0.5% PTMO-coated Q1 was significantly lower than that observed for uncoated Q1, however, with a tendency towards slightly higher mean TIs with time. Nevertheless, good quenching potential and appropriate coating stability was also demonstrated for PTMO at slightly acid conditions.

### *In vivo* determination of coating efficiency and stability

To estimate *in vivo* coating efficiency and stability of the organosilane-based surface coatings, biological activity of pristine and 0.5% PTMO or 0.2% SIVO160-coated Q1 was investigated in rat lungs after intratracheal instillation (3-month post-treatment period). To avoid unspecific overload effects, a relatively low dose (1 mg dust lung^−1^) was selected. There were no effects on body weights in quartz-instilled rats (data not shown), but mean relative lung wet weights of DQ12-treated [5.9 ± 0.66 g kg^−1^ body weight (b.w.); *P* ≤ 0.01] and Q1-treated animals (5.2 ± 0.94 g kg^−1^ b.w.; not statistically significant) were increased 90-day post-treatment, compared to saline-treated controls (4.2 ± 0.83 g kg^−1^ b.w.). Animals exposed to 0.5% PTMO-coated Q1 showed only slightly lower mean relative lung wet weight (4.8 ± 0.59 g kg^−1^ b.w.; no statistical significance) than Q1-treated animals, whereas mean relative lung wet weight of SIVO160-treated animals was clearly reduced to almost control level (4.3 ± 0.53 g kg^−1^ b.w.; no statistically significant difference to saline-treated animals), but without reaching statistical significance with regard to the Q1-treated group.

When evaluating biochemical endpoints in BAL fluid (Table 1A), DQ12 and uncoated Q1 showed the well-known latency period before full display of quartz effects (group mean values on day 90 post-instillation > day 28 post-treatment). Ninety-day post-instillation, pristine Q1 induced 4.2-, 3.8-, and 3.1-fold higher LDH release, β-glucuronidase activity, and total protein, respectively, compared to saline-treated controls. DQ12 was even more potent (11.0-, 18.9-, and 3.8-fold increases). Protective effects of the 0.5% PTMO and 0.2% SIVO160 coatings were clearly evident 28-day post-instillation and were strongly more pronounced for SIVO160. SIVO160 significantly inhibited the Q1-induced increase in LDH release, β-glucuronidase activity, and total protein by 94, 86, and 100%, respectively. Irrespective of its very promising *in vitro* effects, PTMO only slightly reduced Q1-induced LDH release (26%) and increase in total protein (49%). On day 90 post-instillation, significant reduction in Q1-mediated biochemical effects was still evident for SIVO160 (LDH activity: −56%; β-glucuronidase activity: −79%; total protein: −69%). The 0.5% PTMO coating displayed only slight, non-significant inhibition (total protein: −28%).

**Table 1.** *In vivo* determination of coating efficiency and stability. (A) Biochemical endpoints in BAL fluid 28 and 90 days post-treatment. (B) Cell counts in BAL fluid 28 and 90 days post-treatment.

Treatment groups	Dose (mg rat^−1^)	(A) Biochemical endpoints in BAL fluid											
		LDH (U l^−1^)				β-Glucuronidase (U l^−1^)				Total protein (mg l^−1^)			
		Day 28		Day 90		Day 28		Day 90		Day 28		Day 90	
		Mean	SD	Mean	SD	Mean	SD	Mean	SD	Mean	SD	Mean	SD
Physiological saline	0.3 ml	29	12.2	26	12.9	0.20	0.10	0.28	0.15	105	12.3	107	18.7
		—	—	—	—	—	—	—	—	—	—	—	—
Q1	1	82	19.3	108	33.6	0.64	0.28	1.06	0.36	194	22.0	329	63.3
		**	—	n.s.	—	n.s.	—	n.s.	—	n.s.	—	**	—
Q1 + 0.5% PTMO	1	68	22.0	95	13.0	0.64	0.30	0.86	0.29	150	13.0	266	43.3
		n.s.	n.s.	n.s.	n.s.	n.s.	n.s.	n.s.	n.s.	n.s.	°°	*	n.s.
Q1 + 0.2% SIVO160	1	32	6.00	62	21.9	0.26	0.05	0.44	0.11	105	9.7	176	24.6
		n.s.	°°°	n.s.	°	n.s.	°	n.s.	°°	n.s.	°°°	n.s.	°°°
DQ12	1	148	37.7	286	117.2	2.08	1.33	5.28	2.67	291	60.4	409	74.2
		**	—	**	—	**	—	**	—	**	—	**	—
Treatment groups	Dose (mg rat^−1^)	(B) Cell counts in BAL fluid											
		Leukocytes (× 10^3^ ml^−1^)				PMN (× 10^3^ ml^−1^)				Lymphocytes (× 10^3^ ml^−1^)			
		Day 28		Day 90		Day 28		Day 90		Day 28		Day 90	
		Mean	SD	Mean	SD	Mean	SD	Mean	SD	Mean	SD	Mean	SD
Physiological saline	0.3 ml	107	66.2	97	22.4	1	0.5	2	0.8	0	0.0	0.7	0.77
		—	—	—	—	—	—	—	—	—	—	—	—
Q1	1	261	129.1	235	150.8	85	54.2	99	97.5	0	0.0	11.6	8.23
		*	—	n.s.	—	**	—	n.s.	—	—	—	*	—
Q1 + 0.5% PTMO	1	140	24.5	221	106.0	18	11.2	72	34.1	0	0.0	7.9	5.75
		n.s.	n.s.	n.s.	n.s.	n.s.	°°	n.s.	n.s.	—	—	n.s.	n.s.
Q1 + 0.2% SIVO160	1	103	20.5	131	21.5	3	2.1	29	18.5	0	0.0	2.5	1.54
		n.s.	°	n.s.	n.s.	n.s.	°°°	n.s.	n.s.	—	—	n.s.	°°
DQ12	1	353	120.2	651	324.0	105	48.1	249	111.0	0	0.0	11.4	8.45
		***	—	**	—	***	—	**	—	—	—	*	—

Mean, mean value of five animals per group; —, not applicable; n.s., no statistically significant difference. */**/*** significantly different from saline-treated negative controls: *P* ≤ 0.05/0.01/0.001, respectively, Dunnett’s test (left row); °/°°/°°° significantly different from Q1-treated animals: *P* ≤ 0.05/0.01/0.001, respectively, Student’s *t*-test for unpaired values, two tailed (right row). In the Q1-treated group, one animal clearly did not react to quartz treatment with increased cell counts and was thus excluded from statistical analyses with regard to coating efficiency.

On days 28 and 90 post-instillation (Table 1B), DQ12 mediated the strongest increase in total leukocytes and PMNs, followed by Q1 (total leukocytes: 2.4-fold induction 28- and 90-day post-instillation; PMNs: 85.0- and 49.5-fold increase on days 28 and 90, respectively). Additionally, a 16.6-fold (day 90 post-instillation) higher mean lymphocyte count was noted, compared to saline-treated controls. At day 28 post-instillation, the 0.2% SIVO160 coating offered high protection with complete/nearly complete inhibition of Q1-mediated increase in mean leukocyte number (100%) and PMNs (98%). The 0.5% PTMO coating was slightly less effective with 78 and 80% reduction, respectively. On day 90 post-instillation, PTMO nearly lost its protective effect, as indicated by only 10, 28, and 34% inhibition of Q1-induced increase in total leukocytes, PMNs, and lymphocytes. In contrast, SIVO160 still markedly protected lung tissue, with 75% (total leukocytes), 72% (PMNs), and 84% (lymphocyte number) reduction in Q1-mediated increase in cell counts.

### *In vitro* determination of coating efficiency—industrial scale

**Figure 5.**
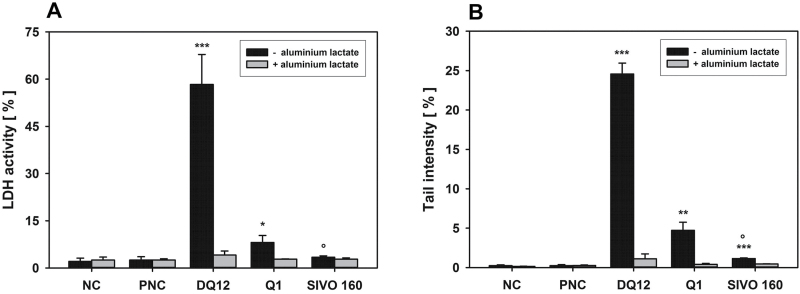
*In vitro* determination of coating effectiveness using Q1 under industrial scale conditions. AM were incubated ± 100 μM aluminium lactate for 4 h with 75 μg cm^−2^ of pristine Q1 or Q1 coated with 0.2% (w/w quartz) SIVO160, Al_2_O_3_ as particulate negative (PNC), or DQ12 as quartz positive control. Culture medium only served as negative control (NC). LDH release (A) and the alkaline comet assay (B) were used to detect membrane and direct DNA damage, respectively. Data represent means ± SD of three independent experiments. */**/***Significantly different from PNC: *P* ≤ 0.05/0.01/0.001, respectively, Student’s *t*-test for unpaired values, two tailed. °Significantly different from pristine Q1: *P* ≤ 0.05, Student’s *t*-test for paired values, two tailed.

Due to promising performance of the 0.2% SIVO160 coating in the *in vivo* 90-day study, the coating step was upscaled for a corresponding production plant, again with Q1 as model quartz. Coating efficiency was subsequently tested *in vitro*. Also under industrial scale conditions, coating with SIVO160 significantly inhibited Q1-mediated LDH release and DNA damage by 84 and 80%, respectively (Fig. 5), without compromising product quality due to integration of the SIVO160-based coating step into the ceramics’ production line (Escrig *et al.*, 2014).

## Discussion

Most likely due to limited i*n vivo* coating stability and/or a reduced availability of already lung-resident quartz particles for curative post-exposure coating, neither PVPNO nor aluminium salts brought a real breakthrough in quartz passivation from the preventive and more importantly curative point of view (Weller, 1975; Zhao *et al.*, 1983; Prügger *et al.*, 1984; Goldstein and Rendall, 1987; Bégin *et al.*, 1993; Dufresne *et al.*, 1994; Bégin *et al.*, 1995), raising the need for more promising preventive quartz-coating strategies for industries with potentially hazardous quartz exposure. In contrast to AL and PVPNO, exhibiting mechanisms based on ionic forces and hydrogen bonding, respectively (Fubini, 1998a, 1998b; Albrecht, *et al.*, 2007), organosilane compounds, with their hydrolysed products organosilanols and organosiloxanes, are able to react covalently with quartz surface silanol groups (Wiessner *et al.*, 1990), envisaging potentially more stable quartz coatings.

Indeed, as shown here, particularly the oligomeric, amino-modified siloxane SIVO160 seemed to possess considerable quartz toxicity-quenching potential and sufficient coating stability and/or potential to enhance quartz clearance from lungs over time. In 1986, Morrison *et al.* investigated the protective potential of a hydrophobic organosilane layer to reduce cytotoxicity of chrysotile asbestos. However, using human pulmonary AM as *in vitro* model, organosilane-mediated decrease in mortality was only observed in the presence of dipalmitoyl lecithin. Later, Wiessner *et al.* (1990) studied the influence of certain organosilane-based coatings on pro-inflammatory and pro-fibrotic effects of α-quartz in the mouse lung after intratracheal injection, addressing well-defined surface charges [i.e. –N(CH_3_)_3_ and –NH_2_ (positive) versus –CH_3_ (neutral)]. Aiming to correlate crystal surface structure characteristics with biological activity, they observed that pulmonary effects of pristine α-quartz were quenched by organosilane surface coatings. Thereby, an –N(CH_3_)_3_ containing organosilane was less effective than organosilanes with –CH_3_ (hydrophobic) and –NH_2_ groups (hydrophilic). Therefore, the authors concluded that electrostatic rather than hydrogen bonding interactions may be primarily responsible for the biological activity of quartz surfaces. But, they did not envisage organosilane-based coatings as a potential preventive approach in industrial settings. First hints towards industrial usage came from Vallyathan *et al.* (1991). They used fresh quartz, coated with the organosilane Prosil 28 (CAS no. 81138-65-0), to investigate effects of fresh versus aged quartz on viability and activity of AM *in vitro*. They convincingly demonstrated a protective effect of Prosil 28, i.e. inhibition of quartz cytotoxicity, hydroxyl radical formation, and, additionally, red blood cell haemolysis, an endpoint which seems to be directly correlated with quartz inflammogenicity (Pavan *et al.*, 2014). The authors hence contemplated to use organosilanes in water sprays of drill bits to reduce quartz hazard in a specific occupational setting. Nevertheless, to our knowledge, organosilane-based coatings to date have not found their way into practical use as preventive agents in quartz-handling industries. Thus, the present study is setting the stage for a new field in occupational hygiene.

After intense physico-chemical characterization of several different organosilane-based coatings and specification of appropriate coating procedures, efficient and concentration-dependent inhibition of Q1-mediated adverse effects by organosilane coatings could be demonstrated *in vitro*, using the commercially available compounds PTMO and SIVO160. This was in line with previous studies (Wiessner *et al.*, 1990; Vallyathan *et al.*, 1991) and further supported the hypothesis of Wiessner *et al.* (1990) that electrostatic interactions might be pivotal to the biologic activity of quartz surfaces. SIVO160, which clearly inhibited adverse effects of Q1, effectively modified the negative, biologically active surface charge of Q1 to net-positivity. An isoelectric or even positive surface charge is thereby supposed to minimize adverse interactions between quartz surfaces and cell membranes.

In the present study, it was possible to passivate both Q1 and DQ12 by the same organosilane-treatment regime, indicating that the developed coating procedures might be universal tools for efficient quartz hazard reduction in the traditional ceramics industry. Nevertheless, the organosilane-to-quartz ratio seemed to be of utmost importance, particularly for SIVO160, as the highest SIVO160 concentration tested (1% w/w of quartz), in contrast to lower ones, was nearly inactive in quenching Q1-mediated effects *in vitro*. SIVO160 represents a pre-hydrolysed, waterborne silane system. As an oligomeric, amino-modified siloxane it possesses highly reactive but stabilized (at low pH) silanol groups. These can react with both silanol groups on the quartz surface, but also with other SIVO160 molecules in a condensation reaction at relatively low curing temperatures, forming a dense network/layer (Evonik, 2009; Mack *et al.*, 2012). It can be speculated that in the environment used, and at higher concentrations, a shift towards condensation and high cross-linking activity may have occurred between the reactive silanol groups and the functional amino groups of the silane system. This may in turn have led to aggregate formation (personal communication: Dr. Burkard Standke, Degussa International AG, Essen, Germany) and hindrance of the quartz-coating reaction. However, it cannot be excluded either that a profoundly altered quartz surface charge may also have been responsible for loss in protective potential at higher concentrations.

After promising *in vitro* results, a confirmatory *in vivo* experiment in rats was performed with 0.5% PTMO- and 0.2% SIVO160-coated Q1. A study design with a 90-day post-treatment observation period was selected to enable evaluation of both coating efficiency and constancy of the surface-protective properties. A protective effect could be demonstrated in rat lungs for both PTMO and SIVO160 on day 28 post-treatment, with markedly higher efficiency for SIVO160. The absolute number of cells was more informative here than relative cell counts. For SIVO160 a marked quartz-quenching effect was still detected 90-day post-treatment, when PTMO had unexpectedly lost its protective properties, indicating a limited potential of the *in vitro* test to really predict *in vivo* long-term tissue reactions.

Marked loss in protective activity of PTMO might be based on slow, time-dependent destruction of the PTMO surface coating. Interestingly, Albrecht *et al.* (2004) presumed partial, mechanistically unclear removal of also PVPNO coating over time *in vivo*, as having observed that a considerable lung-protective effect of PVPNO at least partially persisted over 90 days, however with some adverse effects reappearing at later time points (180- and 360-day post-instillation). As an approach to go more profoundly into conceivable mechanistic details, PTMO coating can be chemically understood as being covalent. An explanation for progressive bondage loss could be a partial disintegration of the particles’ mineral surface under the conditions of lung fluid ambience, i.e. mechanically small debris may be formed or it is simply a dissolution effect by water reaching the surface of the coated quartz particles. Additionally, one can hypothesize that lysosomal enzymes may cause partial removal of coating material with time, as discussed for delayed lung reactivity of some quartz species through delayed removal of Al_2_O_3_ surface contaminations (Adamis *et al.*, 2000). This might, at least in part, explain the obviously higher PTMO coating stability *in vitro* because the acellular AAF incubation approach lacked enzyme activities. Reduced stability of PTMO coating *in vivo* might also be based on avoidance of a high-temperature curing step at 100–200°C to preclude disturbance of the production process and negative impact on product quality by early integration of the coating step into the ceramics production lines. Such a curing step is, however, important for a standard silane system like PTMO to favour condensation. Without an adequate curing step, coating defects and back reactions can occur (B. Standke, Degussa International AG, Essen, Germany, personal communication).

In the lung environment, the markedly higher protective effect of SIVO160, compared to PTMO, may also be caused by different chemistry and a different steric outcome of the coating reaction. While PTMO forms rather ‘hedgehog-like’ coatings, the molecular weight of the SIVO160 molecule has been tailored for optimized wetting behaviour and cross-linking activities, leading to formation of homogeneous cross-linked surface layers (ideally monomolecular films) on substrates. Due to optimized wetting properties, high reactivity, and high cross-linking activity/density through amino functional groups of SIVO160, water penetration through the resulting tight coating layer is prevented, leading to unavailability of potentially free quartz surface silanol groups. Furthermore, SIVO160, in contrast to PTMO, does not need a curing step to build up a tight inorganic–organic network without defects and thereby effectively modifies the quartz surface charge by rendering the surface isoelectric or even positive, with subsequent blunting of biological activity.

Comparable particle size distributions suggest no impact of particle size on observed differences seen in coating efficiency and perhaps stability of SIVO160 and PTMO *in vivo*, but a role of differential modification of particle uptake and clearance cannot be ruled out. Albrecht *et al.* (2007) showed that quartz coating with PVPNO or AL resulted in reduced particle lung burden 90-day post-instillation in rats, compared to pristine quartz. They found lower lung burden with PVPNO-treated quartz than with AL-treated quartz, perhaps due to better and earlier uptake of PVPNO-coated quartz particles compared to AL-coated quartz particles by AM, as observed *in vitro*. These authors concluded that the surface properties of quartz particles are crucial for their uptake by AM, thus dictating their clearance kinetics. As SIVO160-coated quartz particles exhibited a clearly stronger protective effect *in vivo*, compared to the PTMO-coated particles, with differential surface charges and properties of the two materials, it might be hypothesized that the SIVO160 coating perhaps better favoured particle clearance than the PTMO coating. To confirm this working hypothesis, it would be crucial to determine particle lung burdens and the resulting clearance half-times for differentially coated quartz species in future experiments.

## Conclusions

As a progress in industrial hygiene, quartz-induced adverse lung effects in coal industry workers were previously cured by inhalation of protective coating agents (Holt, 1985), used as retroactive risk management tool. In contrary, in the present study, a proactive approach was designed to mitigate quartz toxicity at the beginning of the ceramics production flow. The obtained results show strong evidence for covalent organosilane-based quartz surface coatings being a promising protection strategy to reduce quartz-specific toxicity in the traditional ceramics industry, at least for short- and mid-term periods after occupational exposure. However, considering the human lung with clearance half-times of ~1 year for lung-deposited dusts, further optimization should be pursued, aiming at an *in vivo* stability period in the lung of >1 year. Furthermore, it should be evaluated whether integration of the coating step into ceramics production processes or working with already organosilane-coated quartz raw materials in the production line would provide better and more effective workers’ protection.

## Declaration

The authors declare no conflict of interest relating to the material presented in this article. Its contents, including any opinions and/or conclusions expressed, are solely those of the authors.
